# Adhesion and Proliferation of Osteoblast-Like Cells on Porous Polyetherimide Scaffolds

**DOI:** 10.1155/2018/1491028

**Published:** 2018-11-27

**Authors:** Yanbo Zhang, Ruiyan Li, Wenzheng Wu, Yun'an Qing, Xiongfeng Tang, Wenli Ye, Zhiyong Zhang, Yanguo Qin

**Affiliations:** ^1^Department of Joint Surgery of Orthopedic Center, The Second Hospital of Jilin University, Changchun 130041, China; ^2^Department of Mechanical Manufacturing and Automation, School of Mechanical Science and Engineering of Jilin University, Changchun 130025, China; ^3^Translational Research Centre of Regenerative Medicine and 3D Printing Technologies, The Third Affiliated Hospital of Guangzhou Medical University, 63 Duobao Road, Guangdong 510150, China

## Abstract

The purpose of this work was to investigate the porous polyetherimide scaffold (P-PEIs) as an alternative biopolymer for bone tissue engineering. The P-PEIs was fabricated via solvent casting and particulate leaching technique. The morphology, phase composition, roughness, hydrophilicity, and biocompatibility of P-PEIs were evaluated and compared with polyetherimide (PEI) and Ti6Al4V disks. P-PEIs showed a biomimetic porous structure with a modulus of 78.95 ± 2.30 MPa. The water contact angle of P-PEIs was 75.4 ± 3.39°, which suggested that P-PEIs had a wettability surface. Moreover, P-PEIs provides a feasible environment for cell adhesion and proliferation. The relative cell adhesion capability and the cell morphology on P-PEIs were better than PEI and Ti6Al4V samples. Furthermore, the MC3T3-E1 cells on P-PEIs showed faster proliferation rate than other groups. It was revealed that the P-PEIs could be a potential material for the application of bone regeneration.

## 1. Introduction

In the treatment of critically sized bone defect, tissue engineering scaffold has become the hot research in present bone regeneration [1, 2]. Currently, metals, ceramics, and degradable polymers as well as their composite are the common materials used for the fabrication of tissue engineering scaffolds [3, 4]. However, there are some insufficiencies in each of these materials, such as the mismatched mechanical properties and potential metal ion release in titanium alloys, the brittleness in ceramics, and the insufficient mechanical properties in degradable polymers. The research for new materials in bone tissue engineering proceeded all the time.

Recently, the special engineering plastics applied as biomaterials have attracted much attention. Different from other biopolymers (such as PCL, PLA, and PLGA) [5], the biocompatible special engineering plastics have stronger brace behaviour, and their elastic modulus and stiffness are close to the bone, so that they are qualified for prosthesis or utilization in the large segment of a bone defect at weight-bearing site. PEEK is a typical representative of the special engineering plastics, which had been widely used in orthopedics [6, 7]. Polyetherimide (PEI) is another kind of special engineering plastics with high-temperature stability, corrosion resistance, friction resistance, and sufficient mechanical strength. Meanwhile, it has excellent biocompatibility and the potential application of PEI in biomedical science has been researched, such as hemodialysis [8–10]. Moreover, the PEI is cheaper than PEEK, and it has the similar stiffness to bone and similarity to the physiological structure of bone in charge transfer [11–13]. However, there were fewer researches about the PEI based implant. Particularly, no study reported the biological performance of porous PEI scaffold in bone tissue engineering.

Solvent casting and particulate leaching method was a commonly used method for fabrication of porous polymer scaffold, which is owing to the simple operation, and the pore size and the porosity of scaffolds could be well controlled via the particle size and the amount of the incorporated salt particles [14]. Many literatures have demonstrated that the pore size larger than 300 *μ*m is considered as a suitable dimension for osteocyte ingrowth [15–17]. In this study, porous PEI scaffold (P-PEIs) with interconnected and well-distributed pores (500~600 *μ*m) was successfully fabricated via solvent casting and particulate leaching method. The characteristics of P-PEIs were characterized by SEM and FTIR spectra and the mechanical property was tested by a computer controlled universal testing machine. The* in vitro* biocompatibility of P-PEIs was investigated by MC3T3-E1 cells via SEM, fluorescent staining, and CCK-8 Kit.

## 2. Materials and Methods

### 2.1. Preparation of the Samples

Commercially available PEI rod was purchased from Sigma-Aldrich of USA, and the titanium disks (Ti6Al4V) were purchased from Huitai metal materials Inc. of Dongguan. Both PEI rods and Ti6Al4V disks were cut into disks of Ø 12.5 × 1.5mm and polished with 120-, 300-, 600-, and 1000-grit SIC paper successively. The porous PEI (P-PEI) samples were prepared by melting PEI granules (Sigma-Aldrich, USA). Briefly, The PEI particles granules were dissolved in N,N-dimethylacetamide (Sigma-Aldrich, USA) for 2 h at 130°C with a proportion of 30% w/v. Then the polymer solution was poured into a mold where the NaCl with particle size 500-600 *μ*m had been preloaded and centrifuged at a speed of 4000r/min for 10min. The samples were soaked in distilled water for 48h to ensure the complete separation of NaCl particles from the scaffold. Then the samples were tailored with 12.5 mm in diameter and 1.5 mm in thickness for in vitro test. All of the samples were ultrasonically cleaned in acetone, alcohol, and deionized water.

### 2.2. Chemical and Structural Characterizations

The morphology of P-PEI was observed by E-SEM (XL-30, ESEM FEG Scanning Electron Microscope FEI Company). FTIR characterization was performed on a Nicolet Avatar 360 instrument (Nicolet, Madison, WI) at 25°C. In mechanical property test, cylindrical specimens (n=4) with a diameter of 10 mm and a height of 3 mm were used to evaluate the elastic modulus at a loading rate of 1 mm/min by a universal testing system (Instron5982, USA). Water contact angle of all samples was evaluated using the sessile-drop method with a Krus-DSA30 device. The surface topography and roughness of the samples were measured using an atomic force microscope (AFM, Dimension Icon, Veeco Instruments/Bruker, German).

### 2.3. In Vitro Biocompatibility

#### 2.3.1. Cell Culture

Mouse-derived MC3T3-E1 cells (Procell Life Science & Technology Co., Ltd.) were used for the biocompatibility assays. The cells were cultured in Dulbecco's modified Eagle's medium (DMEM; Hyclone, USA) supplemented with 10% fetal bovine serum (FBS) and 1% antibiotics (100 U/ml penicillin and 100 mg/ml streptomycin) in a humidified atmosphere of 5% CO2 at 37°C. The culture medium was replaced every other day. Cells that reached confluence were used for the following experiments.

#### 2.3.2. Cell Adhesion and Cell Morphology

Cell adhesion and morphology displayed on each group were conducted by CCK-8 Kit, fluorescent staining, and SEM. MC3T3-E1 cells were used for the biocompatibility assays. The cells were cultured in Dulbecco's modified Eagle's medium (DMEM; Hyclone, USA) supplemented with 10% fetal bovine serum (FBS) and 1% antibiotics in a humidified atmosphere of 5% CO_2_ at 37°C. Cell suspension (800*μ*l) at a concentration of 2×10^4^ cells/ml was added to the samples. After 2 h of incubation, the relative adhesion rate was measured by Cell Counting Kit-8 (CCK-8, Dojindo, Japan). In fluorescent staining, the samples were rinsed with PBS, fixed with 4% paraformaldehyde, and stained with 4,6-diamidino-2-phenylindole (DAPI, Sigma, USA) for 5 min. Then, the samples were observed by a fluorescence inverted microscope (IX71, JPN). After 72 h of culture, each sample was rinsed twice with PBS, fixed with glutaraldehyde, and serially dehydrated for cell morphological observation by E-SEM.

#### 2.3.3. Cell Cytotoxicity and Proliferation

The samples were soaked in culture medium for 24 hours at 37°C and then supplemented with 10% fetal bovine serum (FBS) and 1% antibiotics to make the leaching solution for the cytotoxicity. Cell suspension (100ul) at a concentration of 1×10^4^ cells/ml was added onto 96-well plates and cultured for 2 days. Then, the medium was replaced by the leaching solution, and culture medium with or without 5 vol % DMSO was used as a control. After 24h incubation, the mixed solution of CCK-8 and DMEM (10: 100) was added to wells and incubated at 37°C for another 2h. The absorbance of each well was measured at 450 nm.

The proliferation of MC3T3-E1 cells on Ti6Al4V, PEI, and P-PEI samples was evaluated quantitatively by the CCK-8 method. Cell suspension (600*μ*l) at a concentration of 5×10^3^ cells/ml was added to the samples. After 24, 48, and 96 hours' culture, culture medium with 10 vol % CCK-8 solution was added in each well and incubated for another 3 h. The absorbance of each well was measured at 450 nm using a microplate reader (Varioskan Flash, Thermo Scientic). For cytotoxicity, the samples were soaked in culture medium for 24 hours to prepare the leaching solution. Leaching solution with or without 5 vol % DMSO was used as a control. After 24h incubation, each well was added with CCK-8 Kit and incubated for another 2h. The absorbance was measured at 450 nm.

## 3. Results and Discussion

### 3.1. Characterization of the PEI Scaffold

**Figure 1:**
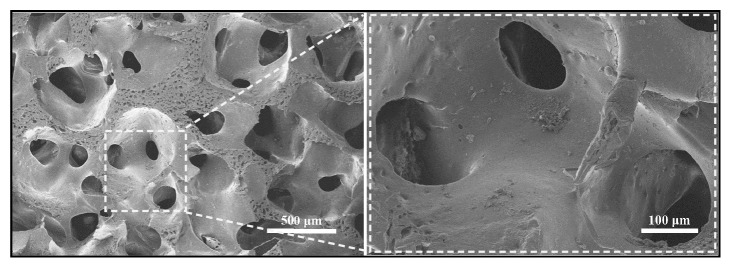
The SEM images of morphologies for P-PEIs.

**Figure 2:**
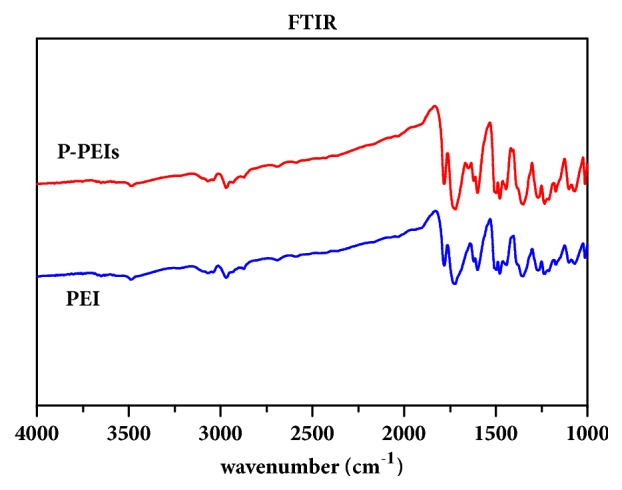
The FTIR spectra for PEI and P-PEIs samples.

Many literatures have demonstrated that the pore size larger than 300 *μ*m is considered as a suitable dimension for osteocyte ingrowth [15–17]. In this study, P-PEIs with pore size range from 500 *μ*m to 600 *μ*m were fabricated. The surface morphologies and internal microstructure of P-PEIs were observed by SEM as shown in Figure 1. It can be seen that the scaffolds exhibited porous structure with evenly distributed and interconnected pores which are regarded as an advantageous factor for the promotion of osteoblast ingrowth and vascularization. The FTIR spectrum was shown in Figure 2 and it can be seen that two samples showed almost overlapping curves. For the typical absorption bands, the absorption peak at approximately 1786 cm^−1^ and 1737 cm^−1^ was for the C=O stretch of imide I, and the absorption peak at 1366 cm^−1^ was for the C−N stretch of imide II. For benzene ring, the absorption peak at approximately 1600 cm^−1^ was observed. The results indicated that the chemical component of P-PEIs was not changed after fabricated and the solvent had been completely removed.

**Figure 3:**
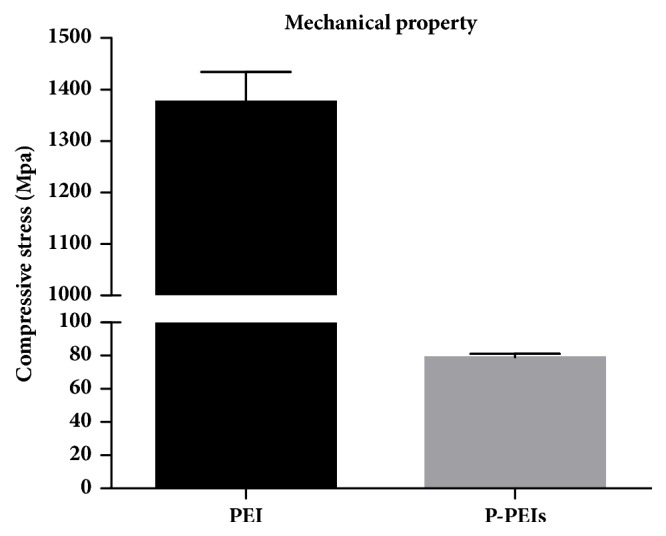
The mechanical property of PEI and P-PEIs samples.

Compression testing was carried out by a universal testing machine to evaluate the mechanical properties of the constructs as shown in Figure 3. The average compressive modulus of PEI dish was 1376.61±57.29 MPa and was significantly lower than Ti6Al4V (≈110 GPa), which could effectively alleviate the side effect of stress shielding caused by the metal implant. Hence, the pure PEI meets the mechanical needs of the implant for bone defect healing in weight-bearing areas. In addition, the modulus of P-PEIs was 78.95±2.30 MPa, falling into the stiffness range of native cancellous bone, which suggests that the P-PEIs can be used for bone defect healing in non-weight-bearing areas [18].

**Figure 4:**
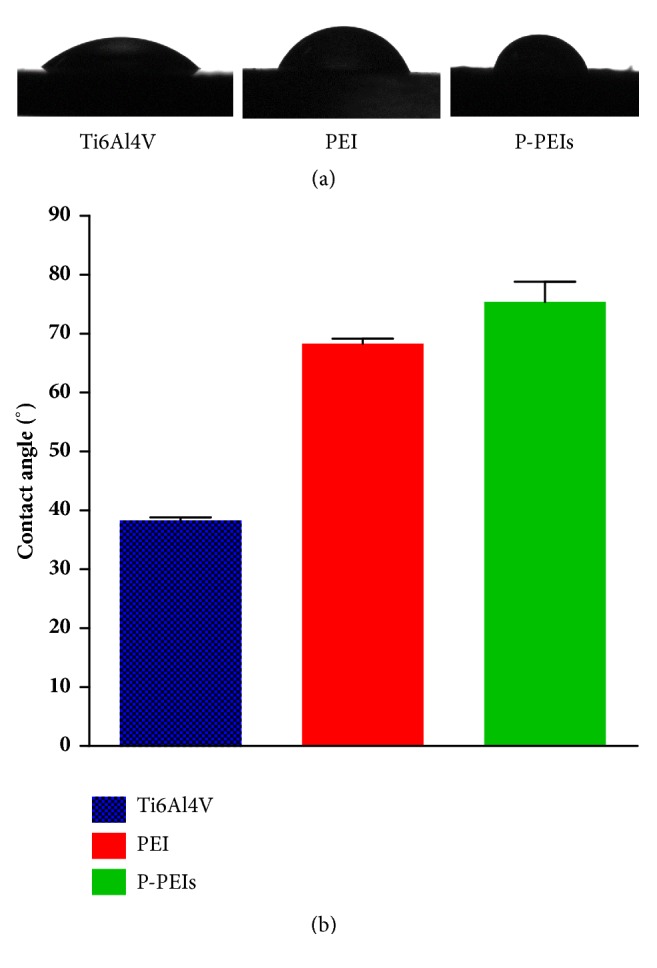
Characterization of the Ti6Al4V, PEI, and P-PEIs samples for the hydrophilicity.

To further understand the properties of different samples, the water contact angle was measured and shown in Figure 4. The average water contact angle of Ti6Al4V sample was 38.3 ± 0.51°, which was smaller than other samples. PEI and P-PEIs samples showed similar values, and they were 68.3 ± 0.84 and 75.4 ± 3.39, respectively. Obviously, all groups had a wettability surface (water contact angle smaller than 90°), while the Ti6Al4V group had better hydrophilicity.

**Figure 5:**
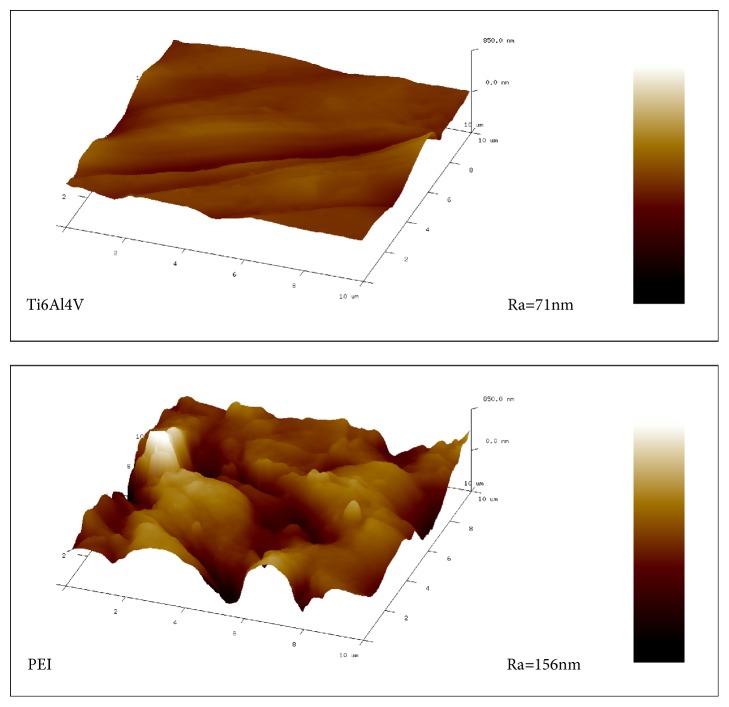
Representative AFM images for the surfaces of Ti6Al4V and PEI samples.

AFM images were captured for further investigation of morphology and roughness of the surface for Ti6Al4V and PEI samples. In Figure 5, the Ti6Al4V surface was relatively smooth, while the surface of PEI was significantly rougher than Ti6Al4V sample. Furthermore, the average roughness (Ra) of PEI sample was 156 nm, which is higher than the Ra of Ti6Al4V (71 nm).

### 3.2. In Vitro Biocompatibility

**Figure 6:**
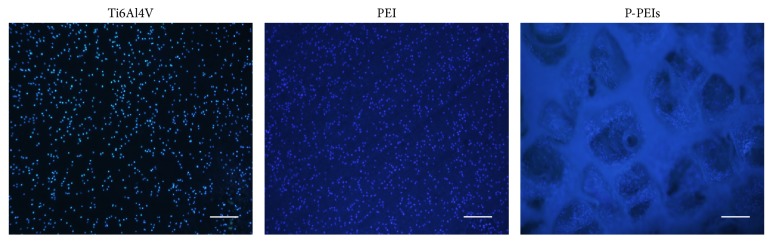
Fluorescent staining of nuclei (blue dots) for the MC3T3-E1 on the samples after 2h incubation. Scale: 500 *μ*m.

**Figure 7:**
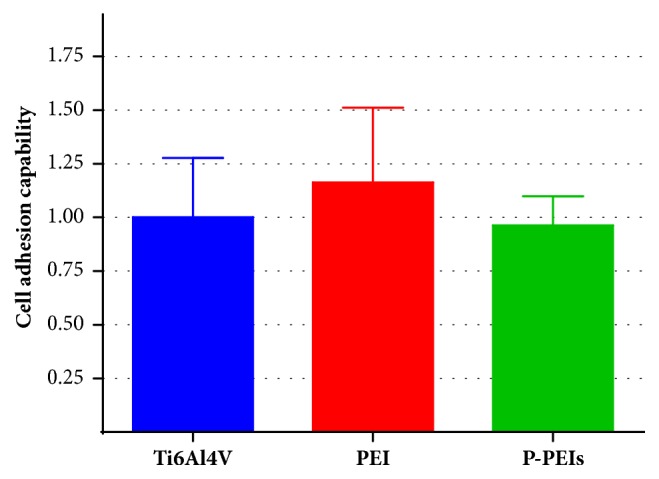
The relative cell capability of all samples after 2h incubation.

Cell adhesion is an important criterion for evaluating the biocompatibility of biomaterials. Figures 6 and 7 show the results of cell amount of MC3T3-E1 cells attached to the different group. From Figure 6, it can be seen that slightly higher amount of blue dots which represent cell nuclei can be observed in PEI dish group compared with the Ti6Al4V group after 2h incubation. However, the cell amount of P-PEIs group was less than PEI dish group. A similar trend can be confirmed by the analysis of relative cell adhesion rate shown in Figure 7. According to the result, obviously, PEI materials had a comparable capacity to promote cell adhesion with Ti6Al4V, and it could be attributed to the rougher surfaces of PEI, whereas the lower amount of cells on P-PEIs was attributed to the porous structure, where the cell suspension was more easily deposited to the bottom of the culture plate through the hole.

**Figure 8:**
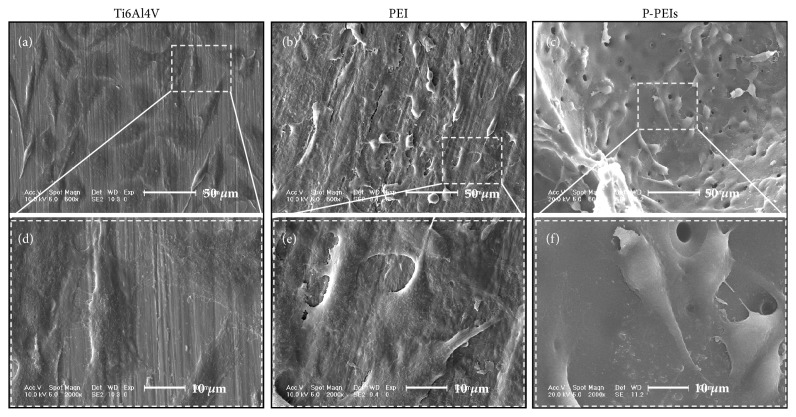
SEM morphology of MC3T3-E1 adhered on Ti6Al4V, PEI dish, and P-PEIs samples after 3 days of culture.

For further inspection, clearly SEM images of MC3T3-E1 cells on the samples are shown in Figure 8; it can be seen that cells on the PEI dish and pore walls of P-PEIs spread well with numerous filopodia compared with the Ti6Al4V group, and cells on P-PEIs displayed obvious better intercellular connection than other groups, indicating that PEI materials provided a better environment for cell attachment and spread. Furthermore, P-PEIs can enhance the interaction of cells.

**Figure 9:**
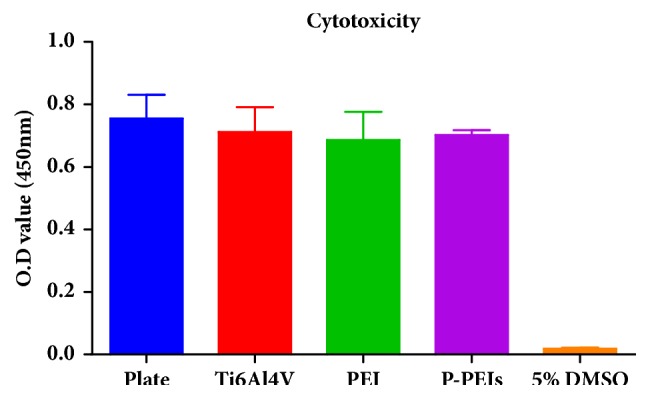
Cytotoxicity assays of Ti6Al4V, PEI, and P-PEIs samples after 24h of incubation.

**Figure 10:**
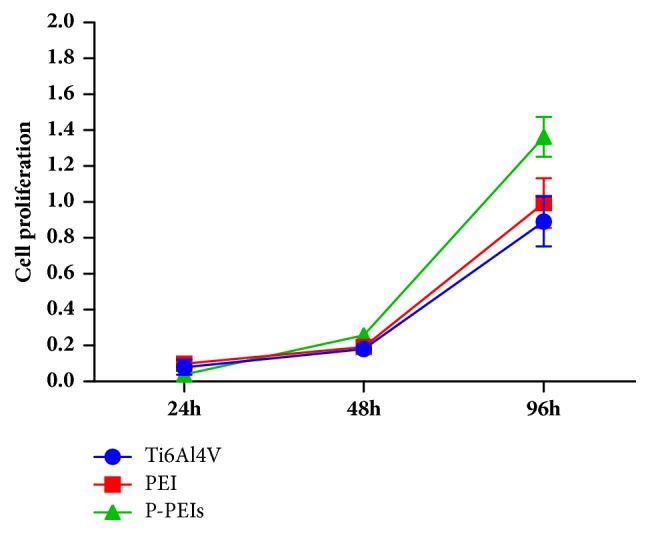
Cell proliferation of MC3T3-E1 cells cultured on Ti6Al4V, PEI dish, and P-PEIs for 24h, 48h, and 96h.

Cell growth on the samples is a long-term and continuous process, and any adverse factors may affect the viability and proliferation of cells. Cell cytotoxicity and viability of the samples were investigated by CCK-8 kit shown in Figures 9 and 10. The cytotoxicity of three groups of samples was tested and shown in Figure 9. The results demonstrated that both PEI dish and P-PEIs were cytocompatible. From Figure 10, it can be seen that there was a similar tendency in the proliferation rate of PEI dish and Ti6Al4V group at each time point, which exhibited a comparable capacity in cell proliferation. The largest amount of cells was observed in P-PEIs group at 96 h, significantly higher than the other two groups, which indicated the highest proliferation rate compared to the previous two groups (P<0.05). Although the relative cell adhesion rate for P-PEIs group is lower than other groups (Figure 7), the proliferation rate of the cells on P-PEIs group is much faster than other groups after 24 h. This is attributed to the three-dimensional structure of P-PEIs, which provided a larger space for cell ingrowth than the other groups.

There are presently numerous works that assess the biological behavior of the alternative biopolymers, which mainly include degradable biopolymers and special engineering plastics. The degradable biopolymers such as PLA, PLGA, and PCL have excellent biocompatibility [5, 19, 20], whereas their weak mechanical properties limit the application. Therefore, some researchers focus on improving the bioactivity and mechanical strength by fabricating the composites of degradable biopolymers and other bioactive materials, such as hydroxyapatite, calcium phosphate, and grapheme [21–23]. However, the degradation rate of composite materials does not match the bone repair process, which would hinder the healing of bone defects. The supporting strength for the implant is very important for the bone defect in the weight-bearing area. The special engineering plastics represented by PEEK applied in medicine have attracted much attention, for the elastic modulus and stiffness are close to the bone. The PEI is a cheaper alternative of PEEK, and it has the similar stiffness to bone and similarity to the physiological structure of bone in charge transfer. The porous PEI scaffold in this work could be a potential material for the application of bone regeneration. However, the surface activity and structure of porous PEI scaffold still need to be improved in future work. Maybe, fabricating composites of PEI and other active materials by 3D printing technology is a feasible research direction.

## 4. Conclusion

In this study, we searched the biocompatibility of PEI materials and successfully developed P-PEIs fabricated by a solvent casting method. The* in vitro* test showed that PEI material had favorable characteristics with preferable cell adhesion, cell growth, and mechanical properties compared to Ti6Al4V. The P-PEIs with interconnected pores which mimic the physiological environment of trabecular bone had provided sufficient space for cell ingrowth and advantageous environment to promote the intercellular connection and interaction of cells, indicating a potential application of P-PEIs as an effective bone graft substitute for the treatment of bone regeneration.

## Data Availability

The data used to support the findings of this study are available from the corresponding author upon request.
